# Attitudes on pharmacotherapy among parents of children with autism spectrum disorders

**DOI:** 10.1192/j.eurpsy.2024.950

**Published:** 2024-08-27

**Authors:** V. Mandic Maravic, M. Vlaisavljevic, S. Lestarevic, J. Vasic, R. Grujicic, M. Pejovic Milovancevic

**Affiliations:** ^1^ Institute of Mental Health; ^2^Faculty of Medicine, University of Belgrade, Belgrade, Serbia

## Abstract

**Introduction:**

Parent-mediated interventions for children with autism spectrum disorder (ASD) have beend recognized as very valuable (1). There is a significant effect of parental attitudes towards treatment on treatment outcomes (2).

**Objectives:**

To evaluate parental attitudes and need for professional support regarding pharmacological treatment of children with ASD.

**Methods:**

We interviewed 67 parents (83.6% mothers) of children with ASD who are regularly treated at our institution. We created a questionnaire with sociodemografic information, clinical characteristics of the child, and parental experience/attitudes on pharmacological treatment.

**Results:**

The average child age was 20.06±4.43; 80.6% were male. The child clinical characteristics and parental sociodemographics are shown Table 1.

**Table 1.** Clinical characteristics of children with ASD/parental sociodemographics

**Clinical characteristics – children**	N	Valid %	X	SD
Speech - 4 words or more	35	52.2		
Epilepsy	13	19.4		
Intellectual dissability	21	31.3		
**Parental sociodemographics**				
Current age of parent (informant)			50.93	6.91
Parent (informant) education Primary and secondary school Attended/finished university or postgraduate degree	25 42	37.3 62.7		
Parent (non-informant) education Primary and secondary school Attended/finished university or postgraduate degree	31 35	47 53

Parental attitudes and feelings when child is treated with medication are shown in Graph 1.

Graph 1. Parental attitudes on medication

We also examined what would help parents in reaching the decision on pharmacotherapy for their children (the results shown in Graph 2).

Graph 2. Parental need of support for decision on medication

In our further analysis, it was shown that the feeling of guilt and helplesness was significantly more present in parents who feared side-effects of medication (p=0.016 and p˂0.001, respectively).

**Image:**

**Figure:**
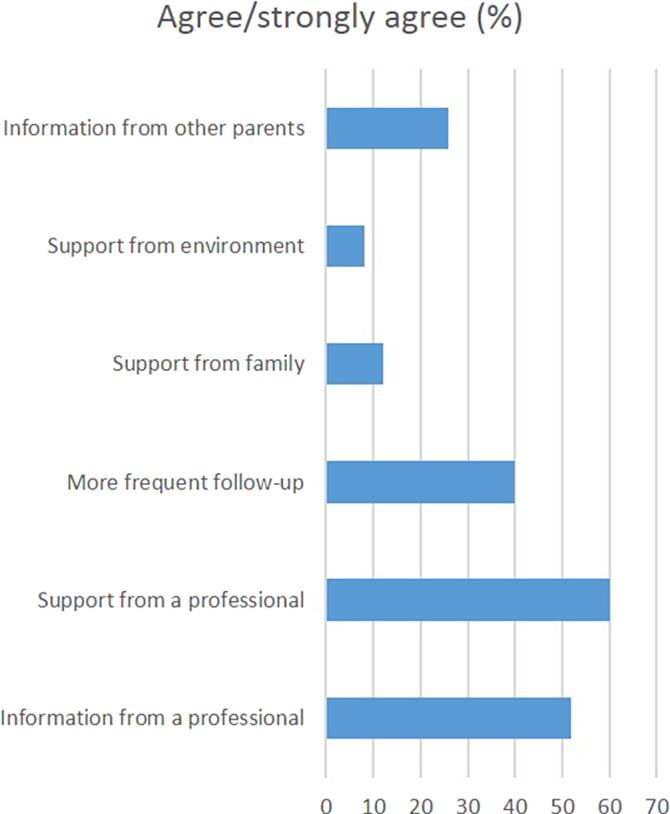

**Image 2:**

**Figure:**
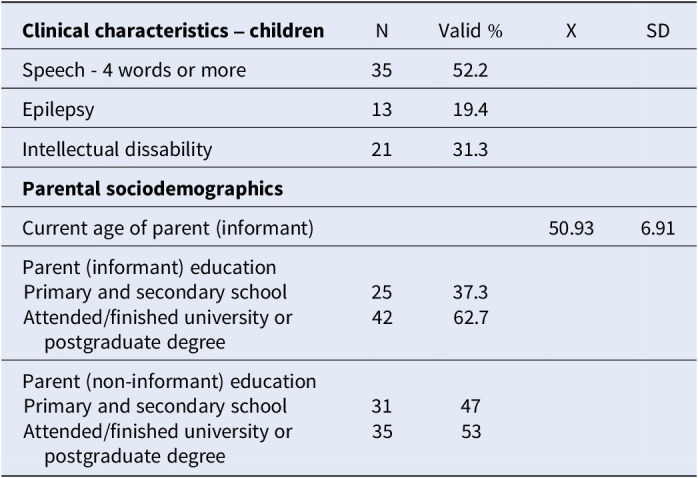

**Conclusions:**

A significant number of parents battle with feelings of helplesness and guilt when medication is introduced in the treatment of their children. There is a great need for information provided by the clinicians as well as psychological support in reaching shared decisions regarding pharmacological treatment of children with ASD.

References:

1. Wang F, Lao UC, Xing YP, Zhou P, Deng WL, Wang Y, et al. Parents’ knowledge and attitude and behavior toward autism: a survey of Chinese families having children with autism spectrum disorder. Transl Pediatr. 2022 Sep;11(9):1445-1457.

2. Hock R, Kinsman A, Ortaglia A. Examining treatment adherence among parents of children with autism spectrum disorder. Disabil Health J. 2015 Jul;8(3):407-13.

**Disclosure of Interest:**

None Declared

